# Clinical Importance of Serum BDNF (Brain-Derived Neurotrophic Factor) Level for the Management of Pregnancies Complicated With Meconium-Stained Amniotic Fluid

**DOI:** 10.7759/cureus.43354

**Published:** 2023-08-11

**Authors:** Nezaket Kadioglu, Umit Yasemin Sert, Nazlı Topfedaisi Ozkan, Sevki Celen

**Affiliations:** 1 Department of Obstetrics and Gynecology, University of Yuksek Ihtisas, Ankara, TUR; 2 Department of Obstetrics and Gynecology, Liv Hospital Ankara, Ankara, TUR; 3 Department of Obstetrics and Gynecology, University of Health Science, Etlik Zubeyde Hanım Education and Research Hospital, Ankara, TUR

**Keywords:** neurotrophins, neonatal outcomes, meconium, fetal distress, bdnf

## Abstract

Objective

The aim of this study was to investigate the association between poor neonatal outcomes and BDNF (brain-derived neurotrophic factor) levels. We aimed to predict the need for an emergency cesarean and prevent unnecessary interventions in cases complicated with meconium-stained amniotic fluid (MSAF).

Methods

This study was designed as a case-control study including three groups. Group A included pregnant women who underwent cesarean due to fetal distress. Group B included the women who delivered vaginally. Groups A and B had cases with the presence of meconium in the amniotic fluid. Group C as a control group had clear amniotic fluid. Demographic features, fetal outcomes, and maternal serum and fetal cord blood BDNF levels (Human BDNF ELISA Kit; Synonyms: ANON2, BULN2; Catalog no: E-EL-H0010 96T) were evaluated.

Results

No significant difference was found between patients with meconium and without meconium in terms of BDNF levels. However, the BDNF level was found to be significantly lower if fetal distress had occurred with MSAF.

Conclusions

In conclusion, the study demonstrated that the level of maternal and fetal cord blood BDNF are both significantly lower when fetal distress occurs with the presence of MSAF.

## Introduction

Meconium is a collection of the digestive tract of a fetus and consists of the contents of intestinal secretions, squamous cell desquamations, lanugo, blood cells, digestive enzymes, bile salts, and up to 80% water [1]. The word ''meconium’’ comes from Greek history and represents the meaning "like opium" [2]. Normally, the passage of meconium occurs in the first 48 hours of birth. 7-22% of normal pregnancies are complicated with meconium passage into the uterine cavity [3]. The clinical significance and the management strategies of meconium-stained amniotic fluid (MSAF) remain different approaches among clinicians. About 5% of pregnancies with MSAF are associated with meconium aspiration syndrome (MAS) which could cause fetal mortality [2]. Accepting the presence of meconium in amniotic fluid as a sign of fetal distress is still controversial. Normally, meconium passage, which needs mature neural control, is a physiological event for term fetuses and cannot be defined as a sign of fetal distress if no other signs are detected (decreased fetal heart rate, fetal movements, scalp blood PH, etc) [4]. However, the presence of meconium in the amniotic fluid can be a sign of fetal distress due to hypoxia and acidosis (resulting in vagal stimulation which triggers peristaltic movements and relaxes anal sphincter) [5]. MAS by aspiration of meconium intrauterine period results in fetal and placental inflammation due to the high level of inflammatory mediators with the excessive bacterial load. Mechanical airway obstruction, surfactant inactivation, and proinflammatory cascade activation have culminated in pulmonary hypertension [6]. The clinical investigations of MAS appear to suggest the typical features of hypoxia which could result in acute respiratory symptoms as well as ischemic encephalopathy [6]. To predict poor neonatal outcome, it is important to know the mechanism underlying intrauterine meconium release and its effects. At this point, it becomes important to know when we need intervention.

Brain-derived neurotrophic factor (BDNF) is a member of neurotrophic factors broadly expressed in the brain [7]. The main role of BDNF is to stabilize neuronal survival by regulating brain development, neuroplasticity, and synaptic connectivity [7, 8]. BDNF is mainly synthesized in the hypothalamus, but BDNF mRNA and proteins are detected in almost all cortical areas in the brain [8]. BDNF and its correlation with oxidative stress and hypoxia have been evaluated in several studies [7, 8]. Alterations of BDNF may have major contributions to neurodegenerative abnormalities, and acute and chronic ischemic processes [7]. In a recent study, Radak et al. showed that there is an adverse relationship between reactive oxygen radicals and BDNF in an experimental model of rats [9]. Similarly, Wu et al. reported that oxidative stress-induced damage is associated with a decreased level of BDNF expression [10]. Chouthai et al. demonstrated that the umbilical cord has different levels of BDNF in different gestational ages and lower BDNF levels were found in preterm fetuses [11]. BDNF production alterations were also shown in intrauterine growth restriction, gestational diabetes, acute respiratory problems of the neonate, and ischemic neonatal brain injury [12]. The pleiotropic effects of neurotrophins such as BDNF were also studied for the effects on airway structure, function, and lung diseases [13]. Recent studies suggest that BDNF might also be a novel marker to predict neonatal hypoxic events during labor [14]. In previous studies, it has been shown that BDNF could pass through the blood-brain barrier, and levels of BDNF are similar in cord blood and fetal brain [15]. Shchelchkova et al. also defined the protective role of BDNF in the case of fetal hypoxia [14].

Because of the mentioned studies claiming the potential interaction between ischemic processes, lung diseases, and BDNF levels, we hypothesized that BDNF levels might have deteriorated in cases complicated with MSAF when the fetus is under distress due to possible hypoxic events. We believe that BDNF levels can be used to predict the need for emergency cesarean section in cases complicated with MSAF and to prevent unnecessary interventions. This article was previously presented as a meeting abstract at the XIV. Turkish German Gynecology Congress which was held between May 28th - June 1th 2022

## Materials and methods

This study was designed as a case-control study and conducted at the obstetrics unit of the Ankara Zekai Tahir Burak Women’s Health Training and Research Hospital. One hundred seventy-six healthy pregnant women were enrolled in the study between May 2017 and April 2018.

All procedures were performed in accordance with the ethical principles of the Declaration of Helsinki and the study was approved by the Ethics Committee of the Ankara Zekai Tahir Burak Women’s Health Training and Research Hospital (protocol number: 7.11.2017/121). Informed consent was obtained before performing the study from all the patients included in the study.

Variables, data sources, and collection

All participants met the following inclusion and exclusion criteria: Pregnant women between the ages of 25-40 years, approved informed consent, and the gestational week between 36 and 40 were included in the study. Those with fetal congenital anomalies, antepartum hemorrhage, multiple pregnancies, with any central nervous system disease, acute or chronic medical illness (gestational diabetes mellitus, diabetes mellitus, hypertension, renal disease, cancer, ongoing infection, history of autoimmune diseases, allergies, drug use, etc.), use of any kind of medication and smoking, and pregnancies after in-vitro fertilization were excluded from the study.

The patients were divided into three groups. Group A included 74 pregnant women who underwent cesarean due to MSAF. The indications for cesarean were fetal distress with documented non-assuring fetal heart rate patterns. Group B included 40 pregnant women who delivered vaginally with MSAF. Group C contained 62 pregnant women and was assigned as the control group with clear amniotic fluid. There was no evidence of fetal distress in the second and third groups.

Demographic data including age, gravidity, gestational week, body mass index (BMI), fetal weight, and sex of the babies were noted. The presence of respiratory distress after birth, fetal exitus, and any kind of sign suggesting an ischemic condition were also noted.

Maternal venous blood sample of about 10 ml was collected in a sterile empty tube without anticoagulant just before birth to evaluate the level of BDNF. Newborn venous blood samples were drawn from the placental part of the umbilical cord before the delivery of the placenta (10 ml). Serum was separated by centrifugation at 3000 rpm for 10 minutes and stored at -70°C until the evaluation.

The serum samples were analyzed by the same technician who was blinded to the status of the subjects. We used a sandwich enzyme-linked immunosorbent assay (ELISA) using the commercially available kit (Human BDNF ELISA Kit; Synonyms: ANON2, BULN2; Catalog no: E-EL-H0010 96T)

Statistical analysis

SPSS Statistics for Windows, version 22.0 (IBM Corp., Armonk, United States) was used for statistical analysis. Data were shown as mean (95% confidence interval) or several cases and (percentage), where applicable. Variables were tested for normality by the Kolmogorov-Smirnov test. The descriptive analyses for normally distributed variables were expressed as the mean ± standard deviation. Categorical variables were expressed as numbers (percentages) and compared with the use of the Chi-square test. We used one-way ANOVA and Kruskal-Wallis H tests for the comparison of multiple groups. The non-parametric post hoc test and the least significant difference test (LSD) test were used for the post hoc analysis. Fetal growth restriction (FGR), intrauterine exitus (IUEX), and respiratory distress of newborns were evaluated to find a relation with BNDF and other clinical and laboratory parameters by multiple logistic regression analysis. Any variable whose univariable test had a P-value < 0.05 was accepted as a candidate for the multivariable model along with all variables of known clinical importance. Adjusted odds ratios and 95% confidence intervals were calculated for each variable, for FGR, IUEX, and respiratory distress of newborns. A P-value less than 0.05 was considered statistically significant.

## Results

A significant difference was not observed in gravidity, age, BMI, and fetal sex between the three groups (p>0.05). Gravidity, gestational week, and fetal weight were significantly different between Group A and Group C (p=0.019, p<0.001, p<0.001, respectively). Fetal weight and gestational week were significantly different between Group B and Group C (p=0.002, p<0.001, respectively) (Table 1). When we evaluated clinical features such as fetal respiratory distress between the three groups, results demonstrated that Group C has significantly lower fetal respiratory distress than Group A and Group B (p<0.001). Group A has significantly higher respiratory distress than Group B (p=0.022). Maternal and fetal cord blood BDNF levels are significantly lower in Group A when compared with Group C (p=0.028, p<0.036 respectively) (Table 1).

**Table 1:** Demographic, clinic and laboratory characteristics of the groups. ¥: inter-group p-value; §: in-group p-value; NS: not significant; BDNF: brain-derived neurotrophic factor

		Group A (n: 74)	Group B (n: 40)	Group C (n: 62)	P-Value ¥	P-Value§
Age		29.48±5.59	28.45±5.84	28.37±5.68	P(AB): NS P(AC): NS P(BC): NS	.822
Gravıdıty		2.27±1.33	2.15±1.23	1.79±0.89	P(AB):.NS P(AC):.019 P(BC): NS	.092
Body Mass Index		30.21±5.06	29.39±4.40	28.66±4.19	P(AB): NS P(AC): NS P(BC): NS	.353
Gestatıonal Week		39.55±1.77	39.87±1.77	40.93±1.51	P(AB): NS P(AC)<.001 p	.629
Fetal Weıght		2660 ±671.99	2709.75±365.63	3327.42±375.10	P(AB): NS P(AC)<.001 p	< .001>
Gender	Gırl	40 (54.1%)	19 (47.5%)	38 (61.8%)	.382	
	Boy	34 (45.9%)	21 (52.5%)	24 (38.7%)		
Fetal Respıratory Dıstress		34 (45.9%)	7 (17.5%)	0	P(AB):.022 P(AC)<.001 p	< .001>
BNDF Level (Maternal)		1702.60±563.72	1889.91±402.20	1948.56±824.73	P(AB): NS P(AC):.028 P(BC): NS	.072
BNDF Level (Fetal Cord)		1605.52±176	1704.89±341	1906.56±435.37	P(AB): NS P(AC):<0.036 P(BC): NS	.077

Table 2 represents the clinical and laboratory features of the births with MSAF and the control group. Gravidity, gestational week, and fetal weight were found significantly different (p=0.043, p<0.001, p<0.001, respectively). When we compared the whole meconium-complicated births to control, BDNF was not different in maternal and fetal cord blood (p=0.080, p=0.064, respectively).

**Table 2:** Demographic and laboratory characteristics of the groups with meconium and without meconium BDNF: brain-derived neurotrophic factor

	Meconium	Control	P-value
Age	29.12 ±5.67	28.37±5.68	.403
Gravidity	2 (1-8)	2 (1-4)	.043
Body Mass Index	29.92±4.84	28.66±4.19	.085
Gestational week	36.66±1.77	37.93±1.51	< .001
Fetal weight	2677.46±703.28	3327.42±375.10	< .001
BNDF level (maternal)	1768.90±48.76	1948.56±105.59	.080
BNDF level (fetal cord)	1666.64±46.4	1906.56±435.37	.064

Table 3 represents the multivariate analysis of perinatal outcomes such as fetal growth restriction and fetal exitus in Group A (cesarean with MSAF). The gestational week and fetal weight were significantly different for both FGR and fetal exitus. BDNF levels were different for FGR (p=0.038 and p=0.046 for maternal and fetal cord blood, respectively).

**Table 3:** Multivariate analysis of perinatal outcomes BDNF: brain-derived neurotrophic factor

	Fetal growth restriction				
	Univariate		Multivariate		
	OR (95%Cl)	P value	OR (95%Cl)	P value	P value
Age	1.005 (.926-1.091)	.900			
Gravidity	1.031 (.731-1.454)	.861			
Body Mass Index	0.997 (.911-1.091)	.945			
Gestational week	0.650 (.478-.884)	.006	0.819 (.570-1.176)	.819	.218
Fetal weight	0.998 (.997-.999)	.001	0.998 (.997-.999)	.002	.125
BNDF level (maternal)	1.001 (1.000-1.002)	.038	1.001 (1.000-.999)	.050	
BNDF level (fetal cord)	976 (938-1000)	.046	987 (954-1001)	.056	

Table 4 shows the multivariate analysis of fetal respiratory problems for age, gravidity, BMI, gestational week, fetal weight, and maternal and fetal cord blood BDNF levels. Fetal weight was found significantly different (p=0.040).

**Table 4:** Multivariate analysis of perinatal outcomes BDNF: brain-derived neurotrophic factor

	Respiratory Distress			
	Univariate		Multivariate	
	OR (95%Cl)	P value	OR (95%Cl)	P value
Age	1.003 (.923-1.089)	.951		
Gravidity	0.994 (.705-1.403)	.973		
Body Mass Index	0.954 (.870-1.047)	.321		
Gestational week	0.727 (.546-.969)	.029	0.856 (.614-1.193)	.360
Fetal weight	0.999 (.998-1.000)	.006	0.999 (.998-1.000)	.040
BNDF level (maternal)	1.000 (1.000-1.001)	.270		
BNDF level (fetal cord)	974 (937-1000)	.360		

## Discussion

The results of our study indicated that there is no significant difference between patients with meconium and without meconium in terms of maternal and fetal BDNF levels. However, the level of maternal and fetal cord blood BDNF was found significantly lower if fetal distress occurred with MSAF. Moreover, it is also conspicuous that fetal growth restriction is significantly related to low maternal and fetal cord BDNF levels according to the results of our study.

Meconium is a consequence of fetal maturation, which is why the incidence is higher among post-term pregnancies. However, we did not detect MSAF during the whole post-term pregnancy. Even some cases with MSAF did not develop fetal distress. Therefore, the crucial question occurs: Is MSAF a result or a reason? What is the reason for different fetal outcomes? This dilemma is the main cause for the need for different markers to improve clinical management and to predict the poor fetal outcome to be able to decide on an intervention.

BDNF recently became a prominent mediator to determine the relationship between neuronal damage after intrauterine adverse events such as fetal hypoxia [14]. The critical role in the protection of neuronal survival causes significant alteration of the level of BDNF [16].

For humans, fetal synthesis, maternal transfer, and placental synthesis are the main areas for the production of BDNF [17]. The factors influencing neuronal survival and development undoubtedly would affect the regulation of circulating BDNF levels starting with the first day of intrauterine life [17]. Gestational age is one of the main contributing factors to the level of BDNF [11]. Especially, the level increases prominently between 29 and 35 weeks of gestation due to the increasing synaptic maturity [11]. In our study, there is a significant difference between the gestational age of Group A and Group C. Although none of the cases was under the gestational age of 35 weeks (which was found to have the maximum increase in the level), the results might be affected by these differences. The cases with MSAF and fetal distress have significantly lower gestational age and BDNF levels. We believe that the difference would be meaningful if the groups had preterm fetuses. In our study, all the groups consisted of fetuses after 36 weeks of pregnancy to eliminate the effect of immature neuronal development on the level of BDNF. Rao et al. Demonstrated that neuronal maturity has a significant effect on BDNF levels among different gestational-aged women [17]. But these results have been obtained by comparison of term and preterm fetuses. We do believe that the BDNF levels of the groups cannot be affected by gestational age since there is no preterm fetus in the three groups. The results of our study indicated that there is a robust difference between BDNF levels of MSAF and the control group if fetal distress occurs. We found that there is no difference between BDNF levels of control and whole MSAF groups (Group A+Group B). This result suggested that BDNF cannot be used to predict MSAF but would be useful to predict fetal distress.

We found that there is a significant difference between the fetal weights of the groups. Antonakopoulos et al. demonstrated that both macrosomia and fetal growth impairment are associated with an increased level of BDNF [18]. According to this study, we would expect to see an increased level of BDNF in Group A and Group B compared to Group C. Decreased levels point to different influential factors except for fetal growth. Mayeur et al. suggested that BDNF has critical roles in implantation, placental development, and function [19]. BDNF is known to have a severe effect on the regulation of angiogenesis, which is essential for placental development [20]. Due to this knowledge, fetal growth restriction is expected to be associated with lower BDNF levels [19]. Similarly, we found that fetal growth restriction is significantly associated with low BDNF levels.

The neonates born through MSAF are almost 100 times more likely to have respiratory problems [21]. When the meconium is aspirated, fills the whole airway unit from the largest airways to the terminal bronchioles [22]. This usually comes out as enhanced airway resistance and deteriorated pulmonary compliance leading to hypoxemia, ischemia, and acidosis [22]. The pathophysiologic pathway is followed by an increase of extracellular glutamate, intracellular calcium, and deprivation of energy providers after a critical level of acidosis [23]. At this point, BDNF is known as a novel molecule used to mend ischemic injuries with its multiple protective effects including anti-apoptosis, anti-inflammation, anti-neurotoxicity, and regenerative role [24]. Animal studies show that BDNF can also be used to improve ischemia-related recovery [24]. It is officially accepted as an effective candidate for defense against ischemic events [25]. In our study, the decreased level of BDNF among the group which had patients with MSAF and fetal distress might be explained with this knowledge corroborating the role of BDNF on the ischemic process. Fetal acidosis can result in fetal distress, ischemic neuronal injury, as well as fetal exitus. We could not find a significant relationship between respiratory problems, fetal exitus, and BDNF levels in our study. However, the results demonstrated that BDNF levels tend to be lower with the presence of fetal distress. All the cases that underwent the cesarean were immediately interfered with by the clinician no sooner than the suspect of fetal distress. We believe that the results might be more significant even if the period of being exposed to acidosis was longer. More in-depth studies with larger series are needed to validate the results of the present study.

## Conclusions

The level of maternal and fetal cord blood BDNF was shown to be significantly lower when fetal distress occurred with the presence of MSAF. To our knowledge, this is the first published study investigating the relationship between BDNF level and MSAF in literature. We believe that studies with larger cases in the future will light the way for the clinical management of MSAF by using serum BDNF alterations.

## References

[1] Ahanya SN, Lakshmanan J, Morgan BL, Ross MG (2005). Meconium passage in utero: mechanisms, consequences, and management. Obstet Gynecol Surv.

[2] Jain PG, Sharma R, Bhargava M (2017). Perinatal outcome of meconium stained liquor in pre-term, term and post-term pregnancy. Ind J Obstet Gynecol Res.

[3] Ziadeh SM, Sunna E (2000). Obstetric and perinatal outcome of pregnancies with term labour and meconium-stained amniotic fluid. Arch Gynecol Obstet.

[4] Nath Nath, P P (2018). Meconium stained amniotic fluid: a correlation with mode of delivery and perinatal outcome. New Ind J OBGYN.

[5] Rawat M, Nangia S, Chandrasekharan P, Lakshminrusimha S (2018). Approach to infants born through meconium stained amniotic fluid: evolution based on evidence?. Am J Perinatol.

[6] Monfredini C, Cavallin F, Villani PE, Paterlini G, Allais B, Trevisanuto D (2021). Meconium aspiration syndrome: a narrative review. Children (Basel).

[7] Hacioglu G, Senturk A, Ince I, Alver A (2016). Assessment of oxidative stress parameters of brain-derived neurotrophic factor heterozygous mice in acute stress model. Iran J Basic Med Sci.

[8] Zhang XY, Chen DC, Tan YL (2015). The interplay between BDNF and oxidative stress in chronic schizophrenia. Psychoneuroendocrinology.

[9] Radak Z, Kumagai S, Taylor AW, Naito H, Goto S (2007). Effects of exercise on brain function: role of free radicals. Appl Physiol Nutr Metab.

[10] Wu A, Ying Z, Gomez-Pinilla F (2004). The interplay between oxidative stress and brain-derived neurotrophic factor modulates the outcome of a saturated fat diet on synaptic plasticity and cognition. Eur J Neurosci.

[11] Chouthai NS, Sampers J, Desai N, Smith GM (2003). Changes in neurotrophin levels in umbilical cord blood from infants with different gestational ages and clinical conditions. Pediatr Res.

[12] Giannopoulou I, Pagida MA, Briana DD, Panayotacopoulou MT (2018). Perinatal hypoxia as a risk factor for psychopathology later in life: the role of dopamine and neurotrophins. Hormones (Athens).

[13] Aven L, Ai X (2013). Mechanisms of respiratory innervation during embryonic development. Organogenesis.

[14] Shchelchkova NA, Kokaya AA, Bezhenar' VF (2020). The role of brain-derived neurotrophic factor and glial cell line-derived neurotrophic factor in chronic fetal oxygen deprivation. Sovrem Tekhnologii Med.

[15] Karege F, Schwald M, Cisse M (2002). Postnatal developmental profile of brain-derived neurotrophic factor in rat brain and platelets. Neurosci Lett.

[16] Husson I, Rangon CM, Lelièvre V (2005). BDNF-induced white matter neuroprotection and stage-dependent neuronal survival following a neonatal excitotoxic challenge. Cereb Cortex.

[17] Rao R, Mashburn CB, Mao J, Wadhwa N, Smith GM, Desai NS (2009). Brain-derived neurotrophic factor in infants &lt;32 weeks gestational age: correlation with antenatal factors and postnatal outcomes. Pediatr Res.

[18] Antonakopoulos N, Iliodromiti Z, Mastorakos G (2018). Association between brain-derived neurotrophic factor (BDNF) Levels in 2(nd) trimester amniotic fluid and fetal development. Mediators Inflamm.

[19] Mayeur S, Silhol M, Moitrot E (2010). Placental BDNF/TrkB signaling system is modulated by fetal growth disturbances in rat and human. Placenta.

[20] Nakamura K, Martin KC, Jackson JK, Beppu K, Woo CW, Thiele CJ (2006). Brain-derived neurotrophic factor activation of TrkB induces vascular endothelial growth factor expression via hypoxia-inducible factor-1alpha in neuroblastoma cells. Cancer Res.

[21] Dargaville PA, Copnell B (2006). The epidemiology of meconium aspiration syndrome: incidence, risk factors, therapies, and outcome. Pediatrics.

[22] Martin RJ, Fanaroff AA, Walsh MC (2014). Fanaroff and Martin's Neonatal-Perinatal Medicine: Diseases of the Fetus and Infant. https://www.us.elsevierhealth.com/fanaroff-and-martins-neonatal-perinatal-medicine-2-volume-set-9780323567114.html.

[23] Szydlowska K, Tymianski M (2010). Calcium, ischemia and excitotoxicity. Cell Calcium.

[24] Chen A, Xiong LJ, Tong Y, Mao M (2013). The neuroprotective roles of BDNF in hypoxic ischemic brain injury. Biomed Rep.

[25] Kingwell K (2010). Neurodegenerative disease: BDNF copycats. Nat Rev Drug Discov.

